# Characteristics and predictors of muscle strength deficit in mechanical ankle instability

**DOI:** 10.1186/s12891-020-03754-9

**Published:** 2020-11-10

**Authors:** Zong-chen Hou, Xin Miao, Ying-fang Ao, Yue-lin Hu, Chen Jiao, Qin-wei Guo, Xing Xie, Feng Zhao, Yan-bin Pi, Nan Li, Zhi-yu Zhang, Dong Jiang

**Affiliations:** 1 Institute of Sports Medicine, Peking University Third Hospital, Beijing Key Laboratory of Sports Injuries, No.49 North Garden Road, Haidian, Beijing, 100191 China; 2 Research Center of Clinical Epidemiology, Peking University Third Hospital, No.49 North Garden Road, Haidian, Beijing, 100191 China

**Keywords:** Chronic ankle instability, Muscle strength deficit, Sports rehabilitation, Limb symmetry index, Predictors

## Abstract

**Purpose:**

Muscle strength training is a common strategy for treating chronic ankle instability (CAI), but the effectiveness decreases for mechanical ankle instability (MAI) patients with initial severe ligament injuries. The purpose of this study was to investigate the characteristics and the potential predictors of muscle strength deficit in MAI patients, with a view to proposing a more targeted muscle strength training strategy.

**Methods:**

A total of 220 MAI patients with confirmed initial lateral ankle ligament rupture and a postinjury duration of more than 6 months were included. All patients underwent a Biodex isokinetic examination of the ankle joints of both the affected and unaffected sides. Then, the associations between the limb symmetry index (LSI) (mean peak torque of the injury side divided by that of the healthy side) and the patients’ sex, body mass index, postinjury duration, presence of intra-articular osteochondral lesions, presence of osteophytes and ligament injury pattern (i.e., isolated anterior talofibular ligament (ATFL) injury or combined with calcaneofibular ligament injury) were analysed.

**Results:**

There was significantly weaker muscle strength on the affected side than on the unaffected side in all directions (*p* < 0.05). The LSI in plantar flexion was significantly lower than that in dorsiflexion at 60°/s (0.87 vs 0.98, *p* < 0.001). A lower LSI in eversion was significantly correlated with female sex (0.82 vs 0.94, *p* = 0.016) and isolated ATFL injury (0.86 vs 0.95, *p* = 0.012). No other factors were found to be associated with muscle strength deficits.

**Conclusion:**

MAI patients showed significant muscle strength deficits on the affected side, especially in plantar flexion. There were greater strength deficits in eversion in females and individuals with an isolated ATFL injury. Thus, a muscle strength training programme for MAI patients was proposed that focused more on plantar flexion training and eversion training for females and those with an isolated ATFL injury.

**Supplementary Information:**

The online version contains supplementary material available at 10.1186/s12891-020-03754-9.

## Introduction

Lateral ankle sprains are a frequently occurring musculoskeletal injury in sports [1]. Approximately 34% of individuals suffer from chronic ankle instability (CAI), which is characterized by a recurrent sprain, episodes of giving-way of the ankle joint, pain, swelling and decreased function [2]. Functional treatment, such as muscle strength training and balance training, is a common strategy for treating CAI with good results, especially for those with grade I and II ligament injuries [3]. Unfortunately, the effectiveness becomes significantly reduced for those with an initial severe ligament injury that develops into mechanical ankle instability (MAI). Approximately 20–40% of those with chronic MAI experience failed to the rehabilitation interventions and is recommended for surgical treatment [4]. The poor effect of rehabilitation may be related to severe deformity of the joint after the ligament is completely ruptured, which may result in excessive muscle strength loss. The conventional muscle strength training programme that applies the same training intensity in each direction of each patient’s ankle joint might not be targeted for those with severe ligament injury and relatively more instability, thereby reducing the training effect. An optimized and more targeted strength training strategy for MAI patients is expected to improve the prognosis of rehabilitation and to reduce the probability of surgery. Compared with mild ankle sprains, these MAI patients may have special muscle strength characteristics and related factors. However, the characteristics and the predictors of muscle strength deficits in MAI patients have rarely been studied.

Most of the reported studies only investigated the overall characteristics of muscle strength in CAI patients without distinguishing functional or mechanical instability, which might be different due to the severity of ligament injury. Even in those studies, the characteristics of the muscle strength deficits in CAI were contradictory. Some have suggested that subjects with CAI showed eversion strength deficits [5], while more recent research using isokinetic dynamometry revealed strength deficits in the invertor musculature [6, 7]. The contradictory results might be due to the relatively small sample size, and the characteristics of the strength deficits in MAI patients are still unknown.

The factors related to ankle sprain recurrence in MAI patients include sex [8], body mass index (BMI) [9, 10], a history of previous ankle injuries [11] and muscle strength deficits. In addition, a muscle strength deficit could be also closely related to the ligament injury pattern and concomitant injuries, which have rarely been studied. In terms of the ligament injury pattern, an isolated lesion of the anterior talofibular ligament (ATFL) occurs in approximately 65% of cases, while combined ruptures of the ATFL and calcaneofibular ligament (CFL) occur in approximately 20% [12]. The isolated ATFL injury or the combined both ligament injuries may affect the degree of lateral instability, resulting in different characteristics of muscle strength. The osteochondral lesions (OCLs) and osteophytes are common concomitant injuries in severe MAI cases, which might also affect walking posture and muscle strength due to the increased joint pain.

The aim of the retrospective study was to explore the characteristics and the predictors of the muscle strength deficits in MAI patients, which could contribute to the development of an optimized muscle training strategy to improve the prognosis of rehabilitation. In the present study, the chronic MAI patients with an initial lateral ankle ligament rupture and postinjury duration of more than 6 months were included. All patients underwent Biodex isokinetic examination of the ankle joints. Then, the associations between the muscle strength deficit and the patients’ demographics, clinical features, ligament injury pattern and concomitant lesions were analysed.

## Methods

### Subjects

From June 2010 to June 2015, 220 CAI patients preparing for arthroscopy and lateral ankle ligament repair operations at our institute were included in the study. All of them were diagnosed with grade III ligament injuries before surgery. Specific inclusion criteria were as follows: aged from 20 to 50; a history of at least one significant ankle sprain; postinjury duration (injury-examination duration) more than 6 months; recurrent sprain (more than two sprains in 6 months) and/or “feelings of instability” [13]; grade III [14, 15] ligament (ATFL or combined CFL) lesion confirmed by both MRI and a positive anterior drawer test (i.e., increased translation of 3 mm compared to the unaffected side or an absolute value of 10 mm of displacement) [16] and talar tilt test (i.e., 10° of absolute talar tilt or a 5° difference compared to the contralateral side) [17].

Exclusion criteria included a history of previous surgeries to the musculoskeletal structures (such as bones, joint structures and nerves) in either lower extremity; bilateral ankle instability; a history of a fracture requiring realignment in either lower extremity; and acute injury to the musculoskeletal structures of other joints of the lower extremity in the previous 3 months [13].

### Data sources and measurement

The data of all the patients were gathered by analysing their admission records, preoperative examinations and surgical records. The basic patient parameters included sex, BMI and postinjury duration. Data such as the presence of OCLs, osteophyte, isolated ATFL injury or combined CFL injury were gathered from the operative records, which were written by both the operator and the assistant at the operation. The OCLs and osteophytes were investigated and described during arthroscopic exploration, and the ligament injury was observed by looking directly at the ligament morphology and exploring its tension with hemostatic forceps. The details of the measurement form were shown in the supplementary file. The study was approved by the IRB Medical Committee of our hospital (IRB00006761–2016011).

### Muscle strength measurement

As described in TW Kaminski’s research [18], isokinetic strength was assessed with a Biodex isokinetic dynamometer (Biodex Medical Systems Inc., Shirley, NY). Each subject was seated on the Biodex chair, with the hip angle 80^°^ flexion (0^°^ neutral position), and a knee pad was placed under distal femur and secured with a strap allowing for approximately 20^°^ to 30^°^ of knee flexion, then the foot was securely fastened into the ankle inversion/eversion or plantar flexion/dorsiflexion footplate attachment. Once positioned, the participant’s active range of motion was used to determine the start and stop angles. Each patient performed the concentric contraction mode at 60 and 120°/s on both ankles. To become familiar with the isokinetic test procedure, each subject was allowed three submaximal (50% capacity) warm-up repetitions at each velocity. Then, the order of the isokinetic test velocity (60°/s or 120°/s) and ankle motion (plantar flexion, dorsiflexion, eversion or inversion) was randomized using a coin flip to minimize any potential learning effects. Three maximal concentric test repetitions were completed at both test velocities under researcher’s encouragement: “move your ankle in the plantarflexion-dorsiflexion/eversion-inversion direction as fast and hard as you could”. Repetitions were repeated if the torque curve did not closely match the previous attempt to ensure that the subject was exerting maximal effort with each test repetition. The resting interval was approximately 1 min between tests for each motion, velocity, and side. At the end of testing, peak torque data were extracted from the torque curves, and the mean peak torque was used to calculate the “limb symmetry index” (LSI), which was defined by the mean peak torque of the injured side divided by that of the healthy side. The LSI and mean peak torque data were then subjected to statistical analysis.

### Variables and statistical analysis

All variables were tested for normality using the Shapiro-Wilk test. Muscle strength was measured and compared between the involved and intact ankles using paired-examples t tests. Comparison of the LSI in different directions and with different velocities was performed using the Mann-Whitney U test. To identify the factors related to muscle strength deficits, the Mann-Whitney U test and Spearman correlation coefficient were calculated between each potential factor described above and the LSI. A two-tailed *p* value < 0.05 was considered statistically significant.

## Results

### Participant characteristics

**Fig. 1:**
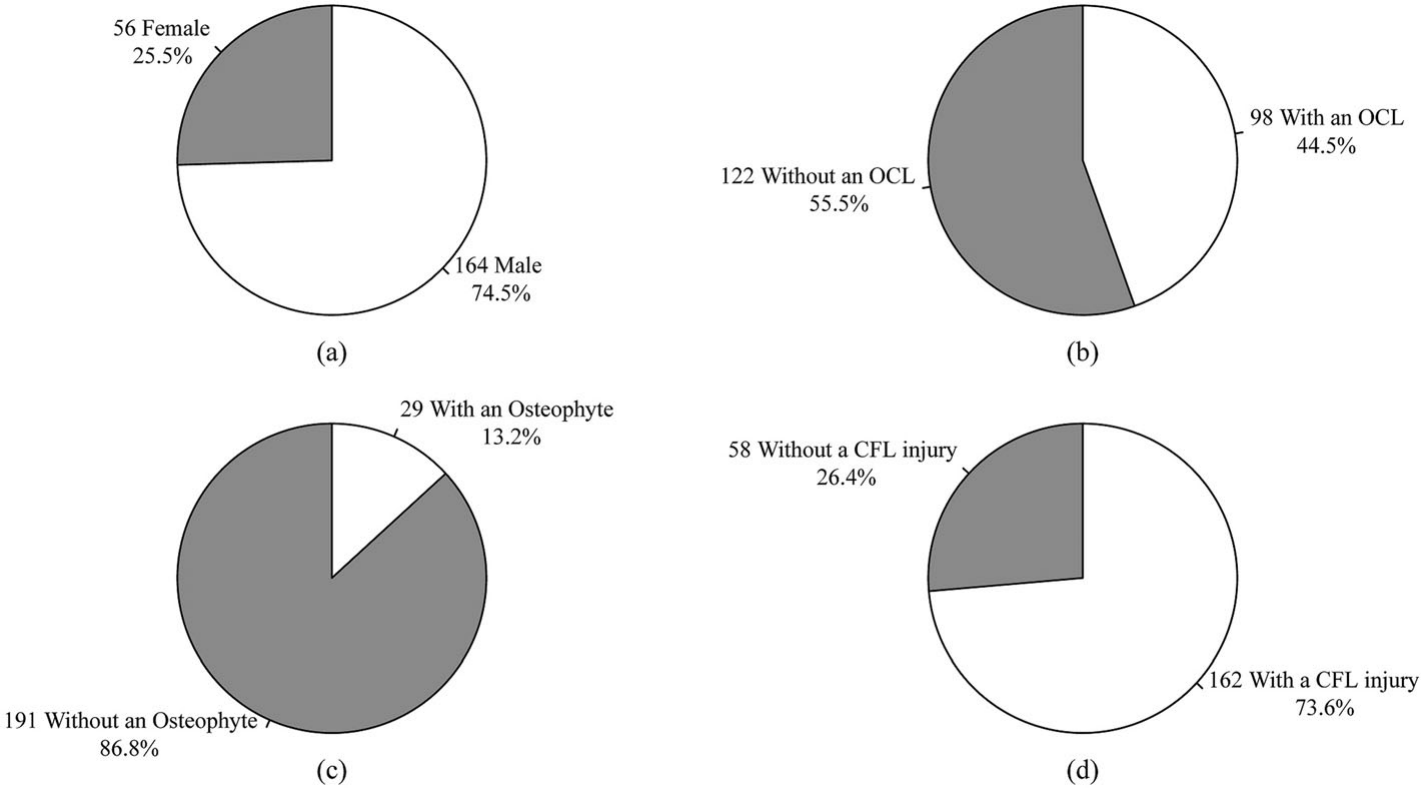
Baseline patient information and injury data. OCL, Osteochondral lesion; CFL, Calcaneofibular ligament

Two hundred and twenty participants (164 male and 56 female) were included and analysed in this study. The mean age was 33.92 ± 4.90 years, the mean postinjury duration was 33.16 ± 41.83 months, and the mean BMI was 24.81 ± 3.78 kg/m^2^. The characteristics of the participants are presented in Fig. 1.

### Peak torque

As shown in Table 1, compared to those on the unaffected side, all the mean peak torques on the affected side were significantly lower in each direction at two different velocities (*p* < 0.05).

**Table 1:** Comparison of the mean peak torque on both sides at two different velocities

Peak torque (N/kg)	Affected side	Unaffected side	T values	*p*
120°/s PF	0.40 ± 0.17	0.45 ± 0.18	−5.841	< 0.001^a^
120°/s DF	0.20 ± 0.08	0.23 ± 0.08	−4.206	< 0.001^a^
120°/s IV	0.23 ± 0.11	0.27 ± 0.11	−6.946	< 0.001^a^
120°/s EV	0.24 ± 0.11	0.28 ± 0.12	−5.910	< 0.001^a^
60°/s PF	0.48 ± 0.22	0.54 ± 0.21	−2.332	0.02^a^
60°/s DF	0.23 ± 0.10	0.26 ± 0.09	−3.359	< 0.001^a^
60°/s IV	0.24 ± 0.11	0.28 ± 0.12	−6.789	< 0.001^a^
60°/s EV	0.25 ± 0.11	0.29 ± 0.13	−6.532	< 0.001^a^

*DF* Dorsiflexion, *PF* Plantar flexion, *IV* Inversion, *EV* Eversion

^a^represents a significant difference

### Limb symmetry index for different velocities and directions

**Fig. 2:**
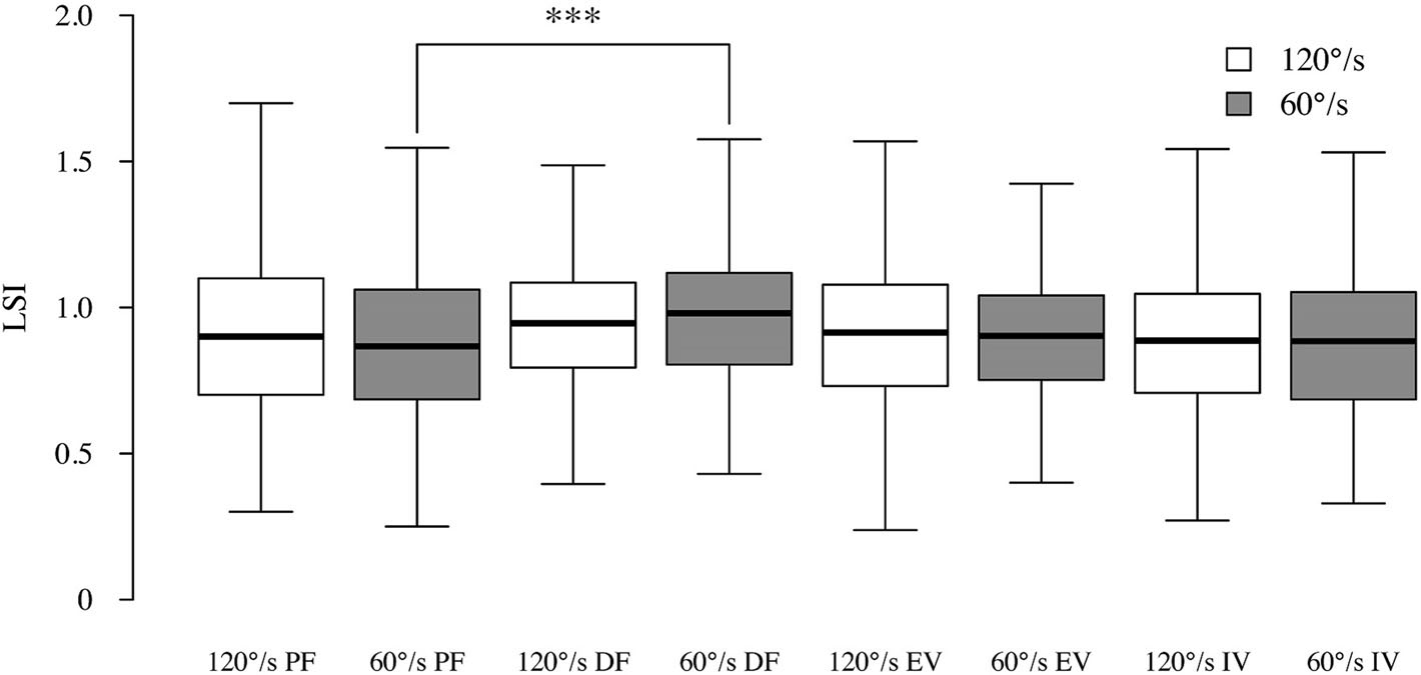
Comparison of the limb symmetry index for different directions and different velocities. The LSI at 60°/s in plantar flexion was significantly lower than that in dorsiflexion. LSI, limb symmetry index; DF, dorsiflexion; PF, plantar flexion; IV, inversion; and EV, eversion. *** Significant difference with *p* < .001

The results for the median LSI for different directions and different velocities were shown in Fig. 2. When comparing the LSI by velocity, only those at 60°/s in plantar flexion and dorsiflexion (0.87 vs 0.98, *p* < 0.001) showed a significant difference. No significant differences were found for any other directions or velocities.

### Correlation analysis for the LSI

**Fig. 3:**
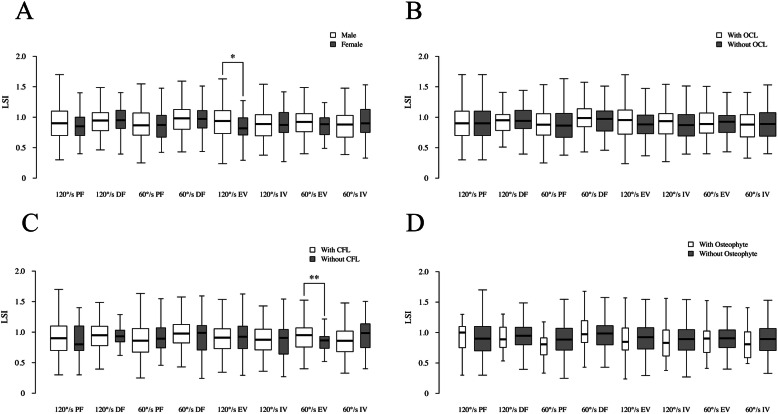
Univariate analysis for sex, CFL injury, presence of an OCL and presence of osteophytes. A, B, C, D represent the comparison between groups divided by sex, CFL injury, presence of an OCL and presence of osteophytes, respectively. Female sex and an intact CFL showed a significant correlation with a lower LSI in eversion. *Significant difference with *p* < .05. ** Significant difference with *p* < .01

The results of the Wilcoxon signed rank test and Pearson’s test are shown in Fig. 3; they present the correlations of the LSI and the categorical variables including sex, OCL (or not), osteophyte (or not), isolated ATFL injury or combined CFL injury. The results indicated that the 120°/s EV LSI in the female group was significantly lower than that of the male group (0.82 vs 0.94, *p* = 0.016), and the 60°/s EV LSI of the patients with an intact CFL was significantly lower than that of those with a combined CFL injury (0.86 vs 0.95, *p* = 0.012). The results for the continuous variables, including BMI and postinjury duration, are shown in Table 2 with no significant correlations (*p* > 0.05).

**Table 2:** Univariate analysis of BMI and injury-examination duration

LSI	BMI		Injury-examination duration	
	r	*p*	r	*p*
120°/s PF	−0.004	0.957	−0.046	0.516
120°/s DF	−0.021	0.768	−0.044	0.534
60°/s PF	−0.004	0.956	−0.021	0.763
60°/s DF	−0.066	0.348	−0.094	0.181
120°/s EV	−0.029	0.683	−0.002	0.980
120°/s IV	−0.060	0.393	−0.101	0.152
60°/s EV	−0.026	0.712	−0.051	0.474
60°/s IV	−0.113	0.111	−0.092	0.195

## Discussion

According to the results of the present study, there was significantly weaker muscle strength on the affected side in individuals with MAI. Greater muscle strength deficits were shown in plantar flexion than in dorsiflexion at a velocity of 60°/s, which was not influenced by the testing velocity. The female and isolated ATFL injuries were significantly related to a lower LSI in eversion and a greater eversion strength deficit.

The existence of an ankle muscle strength deficit on the affected side was similar to the results of other studies reporting deficits in dorsiflexion [19], plantar flexion [20], inversion [7] and eversion [7]. A prospective study [19] showed that a dorsiflexion muscle strength deficit was an intrinsic factor for individuals with inversion ankle sprains, and the individuals with ankle instability showed a weaker dorsiflexion muscle strength than the healthy individuals. Phillip A Gribble [20] also found that individuals with CAI exhibited significantly weaker plantar flexion strength in their injured limb than in their non-injured limb. However, for those studies, individuals with severe MAI with grade III ligament tears have not been isolated from among all CAI patients, which may limit the application of conclusions in the clinical practice. Our results indicated that MAI patients present a similar muscle deficit to CAI patients with weaker strength in all directions on the injured side.

General CAI strength training emphasizes dorsiflexion muscle strength to compensate for lateral ankle instability caused by the initial ligament rupture [21, 22]. On the other hand, the results of the present study showed that the LSI at 60°/s in plantar flexion was significantly lower than that in dorsiflexion (0.87 vs 0.98, *p* < 0.001), indicating that the muscle strength deficit in plantar flexion was more serious than that in dorsiflexion for MAI patients. Gribble also found that patients with CAI showed a significant muscle strength deficit in plantar flexion but not in dorsiflexion [20]. The differences might result from the test indicators; Gribble researched the peak torque, while we researched the LSI. In addition, it is noteworthy that the testing velocity did not affect the muscle strength deficit of the mechanical instability individuals. A meta-analysis found that there were no differences between the < 110°/s group and the > 110°/s group in terms of concentric eversion strength [23]. Therefore, the muscle deficit tends to be more serious in plantar flexion and not related to velocity.

In terms of the predictors, a significantly lower LSI at 120°/s in eversion was evident for females than for males (0.82 vs 0.94, *p* = 0.016), which indicated a greater ankle eversion strength deficit for the females. Hosea et al. [24] also found that compared with male athletes, female athletes were at 25% increased risk of suffering a grade I ankle sprain. Compared with men, women showed a significantly increased rate ratio for ankle sprain of 1.83 (95% confidence interval, 1.52–2.20) [8]. Although not directly noted, the tendency of sprain recurrence in women could be related to the relatively weaker muscle strength. More serious eversion strength deficits may be the cause of higher sprain risk in the female athletes. Future research could focus on whether sex differences are activity-specific and thus related to training behaviours or whether the difference in risk is related to anatomical or physiological sex differences.

Interestingly, the patients with isolated ATFL injuries showed significantly lower LSI at 60°/s in eversion (0.86 vs 0.95, *p* = 0.012) than the patients with combined CFL injuries, which might differ from what is generally assumed. A previous study showed that the CFL accounted for 50–70% of complex ankle joint stability during inversion, especially in dorsiflexion [25]. As an important structure for maintaining ankle varus and subtalar joint stability, the injury of calcaneal and fibula ligaments will significantly increase the joint relaxation. Therefore, it seems that MAI individuals combined with a CFL injury have weaker eversion strength and more ankle laxity. The interesting results of the present study might be attributed to the additional CFL injury contributing to the compensated increase in eversion strength to account for the instability of inversion activities. The mechanism of this interesting finding of the present study needs further biomechanical or kinematic studies.

To our knowledge, this is the first study to evaluate the characteristics of individuals with MAI and to explore the potential predictors to the LSI. The most prominent strength of the present study is the relatively large sample size with complete and accurate information for all the demographics and clinical features. The ligament injury pattern and concomitant lesions were obtained from medical records and confirmed by intraoperative evaluation. The integrity of those factors could help us to better analyse the correlations with muscle strength deficits for MAI patients with initial severe ligament injuries.

There were still some limitations of the present study. Firstly, all the patients included were ready to undergo surgery and thus had complicated and uncontrollable treatment backgrounds prior to enrolment, limiting the applicability of the conclusion of the present study. Secondly, although this study has covered the patient’s clinical features and injury data, there are still some factors (the number of sprains of the patient, daily activity, the previous rehabilitation, etc.) have not been analysed. Thirdly, the ligament injury pattern only incorporated ATFL and CFL injuries, the effect of other stabilizing structure (deltoid ligament, syndesmosis, etc.) need further study.

## Conclusion

The MAI patients showed significant muscle strength deficits on the affected side, especially in plantar flexion. Greater strength deficits in eversion were shown in females and individuals with isolated ATFL injury. Thus, a muscle strength training strategy for MAI patients was proposed to focus more on plantar flexion training and eversion training for females and those with isolated ATFL injuries.

## Supplementary Information

**Additional file 1.**

## Data Availability

The datasets used and/or analysed during the current study are available from the corresponding author on reasonable request.

## Abbreviations

CAI: Chronic ankle instability
MAI: Mechanical ankle instability
LSI: Limb symmetry index
BMI: Body mass index
ATFL: Anterior talofibular ligament
CFL: Calcaneofibular ligament
PF: Plantarflexion
DF: Dorsiflexion
IV: Inversion
EV: Eversion
OCL: Osteochondral lesion
