# Supplementary material for: Characteristics and predictors of muscle strength deficit in mechanical ankle instability
Source: BMC Musculoskelet Disord. 2020 Nov 10;21:730. doi: 10.1186/s12891-020-03754-9 (PMC7654059; doi:10.1186/s12891-020-03754-9)
Supplement: Supplementary file 1 — Additional file 1. [file 12891_2020_3754_MOESM1_ESM.docx]

**Basic information:**

Name:

Age:

Sex:

Height:

Weight:

BMI:

**Clinical assessment information:**

MRI diagnosis: injury of the anterior talofibular ligament (ATFL) and the calcaneofibular ligament (CFL), osteochondral lesions (OCLs), other lesions

Physical examination:

Anterior drawer test: I°, II°, III°

Talar tilt test:

**Ankle sprain related information:**

Injury history:

Numbers of sprain:

Duration since first sprain (month):

**Evaluation of the ligament injury type at operation:**

Isolated ATFL injury:

Isolated CFL injury:

Both ligament injury:

**Evaluation of the combined damage at arthroscopy:**

The OCLs

Osteophytes

**Isokinetic muscle strength information:**

Peak torque/weight(N/kg)

60°/s plantar flexion:

60°/s dorsiflexion:

60°/s eversion:

60°/s inversion:

120°/s plantar flexion:

120°/s dorsiflexion:

120°/s eversion:

120°/s inversion:

Limb symmetry index

60°/s plantar flexion:

60°/s dorsiflexion:

60°/s eversion:

60°/s inversion:

120°/s plantar flexion:

120°/s dorsiflexion:

120°/s eversion:

120°/s inversion:
